# Examining Diversity in Digital Therapeutics Clinical Trials: Descriptive Analysis

**DOI:** 10.2196/37447

**Published:** 2023-08-02

**Authors:** Joel Adu-Brimpong, Jennifer Pugh, David Agyen Darko, Lisa Shieh

**Affiliations:** 1 School of Medicine Stanford University Stanford, CA United States; 2 Division of Dermatology Washington University School of Medicine St Louis, MO United States; 3 Department of Emergency Medicine University of Texas Southwestern Medical Center Dallas, TX United States

**Keywords:** digital therapeutics, DTx, clinical trials, health informatics, digital health, digital medicine, DTx clinical trials, health equity, digital therapy, demographic, sociodemographic, representation, software, telehealth, telemedicine

## Abstract

**Background:**

Digital therapeutics (DTx) are an emerging class of software-based medical therapies helping to improve care access and delivery. As we leverage these digital health therapies broadly in clinical care, it is important to consider sociodemographic representation underlying clinical trials data to ensure broad application to all groups.

**Objective:**

We review current sociodemographic representation in DTx clinical trials using data from the Digital Therapeutics Alliance Product Library database.

**Methods:**

We conducted a descriptive analysis of DTx products. We analyzed 15 manuscripts associated with 13 DTx products. Sociodemographic information was retrieved and compared with the US population’s demographic distribution.

**Results:**

The median study size and age of participants were 252 and 43.3 years, respectively. Of the 15 studies applicable to this study, 10 (67%) reported that females made up 65% or greater of the study cohort. A total of 14 studies reported race data with Black or African American and Asian American individuals underrepresented in 9 and 11 studies, respectively. In 7 studies that reported ethnicity, Hispanics were underrepresented in all 7 studies. Furthermore, 8 studies reported education levels, with 5 studies reporting populations in which 70% or greater had at least some college education. Only 3 studies reported health insurance information, each reporting a study cohort in which 100% of members were privately insured.

**Conclusions:**

Our findings indicate opportunities for improved sociodemographic representation in DTx clinical trials, especially for underserved populations typically underrepresented in clinical trials. This review is a step in examining sociodemographic representation in DTx clinical trials to help inform the path forward for DTx development and testing.

## Introduction

Digital health is here to stay. There is an undeniable place for technological advancements in the field of medicine as society continues to incorporate technology into almost every facet of life. Health care is no exception; it has already been augmented with the use of medical health applications, remote patient monitoring, and wearable technologies increasingly mediated by artificial intelligence. The advancements of today are merely a glimpse into the innovations of the future. These developments ultimately have immense potential to reduce or exacerbate current disparities. The question becomes who will benefit and who will be left out. Traditional obstacles to health and wellness such as cultural differences, educational background, transportation, insurance, housing security, and age have the ability to persist and take new form. As medicine continues to be shaped by the technological era, it is important that digital technologies be engineered with diverse populations in mind. Therefore, it is crucial that these populations be involved in early scientific investigations to assure that such benefits are relevant and equitably distributed for the optimization of care delivery and health outcomes. Within the broad spectrum of digital health technology is the nascent area of digital therapeutics (DTx), which are software-based medical therapies developed to aide in the prevention, management, and treatment of chronic and acute diseases [1]. For example, Somryst is a mobile app–based, prescription-only digital therapeutic, created by Pear Therapeutics, which packages cognitive behavioral therapy strategies using software to help treat adult patients experiencing chronic insomnia [2]. These software-based therapies are unique as they require scientific validation through clinical trials. These clinical trials investigate the usability, accessibility, sustainability, and overall benefit among different populations [1,3].

The evidence-based nature of these emerging therapeutics bolsters the potential for these software-mediated technologies to become a standard part of clinical care in the very near future. In fact, MassHealth, the Medicaid and the Children’s Health Insurance Program of Massachusetts, recently announced plans to reimburse for prescription DTx [4-6]. DTx can be prescribed as a monotherapy or as a complement to traditional pharmacotherapy [3]. Current DTx target a host of chronic diseases (eg, diabetes, asthma, and depression) that disproportionately affect vulnerable and medically underserved populations, such as racial and sexual minorities, older adults, individuals from low-income households, those without higher education, and those with disabilities. With the introduction of the Medicaid and the Children’s Health Insurance Program access to prescription Digital Therapeutics Act to Congress in December 2022, the CEO of the Digital Therapeutics Alliance (DTA), Andy Molnar, was quoted as saying that DTx hold particular value for the Medicaid population as they are convenient, accessible, and provide personalized treatment options to address many unmet medical needs [4,6]. The past has proven that access to care is more complicated than providing resources and that it is challenging to theorize benefits without assuring ease of access, usability, and overall benefit to the most vulnerable populations. According to a study published in *The Lancet Regional Health – Americas* that analyzed US clinical trials between 2000 and 2020, only 43% reported any race or ethnicity enrollment data [7]. Yet, minorities are included in the populations predicted to benefit most from these novel innovations.

As DTx products continue to proliferate, one key factor in improving access to, engagement with, and proper use of emerging digital health therapies, particularly for vulnerable and medically underserved populations, will be the representation of these groups during clinical trials. DTx are fundamentally different from traditional pharmaceuticals such as oral medications. Sustained engagement and the ability to interact with these software-based medications are vitally crucial. As a result, the education, socioeconomic status (SES), literacy, and cognition among other features noted in this paper may be even more vitally important when it comes to developing an inclusive DTx clinical trial cohort than traditional pharmaceuticals to ensure access, engagement, and proper use of DTx, enabling benefits for all groups. Indeed, recent publications have emphasized the need for increased demographic representation, and data reporting, early in the development, testing, and implementation of emerging digital health technologies to ensure the maximum impact for populations affected [7,8]. As DTx are still nascent, this provides a critical opportunity to monitor the development and access to these therapies in accordance with digital health equity recommendations and principles, particularly in clinical trials. In this report, we review the sociodemographic metrics and population representation currently reported in DTx clinical trials.

## Methods

### Study Selection Criteria

We performed a review of DTx products listed in the Product Library of the DTA web page from December 8, 2021, to December 15, 2021. The DTA is based in the United States and was founded in 2017 as the leading, not-for-profit advocacy group composed of industry and thought leaders focused on the promotion, education, and advancement of DTx [1]. The DTA Product Library is a publicly available database [9]. Although the DTA Product Library does not contain an exhaustive list of all current DTx products, among the products featured are those from DTx companies that have signed or agreed to the 10 foundational principles and best practices of DTx. We used the DTA database as it provides an emerging robust repository of validated DTx tools that have at least 1 randomized controlled trial (RCT) published on the technology or abide by the following DTx best practices and guidelines as outlined by DTA: (1) prevent, manage, or treat a medical disorder or disease; (2) produce a medical intervention that is driven by software; (3) incorporate design, manufacture, and quality best practices; (4) engage end users in product development and usability processes; (5) incorporate patient privacy and security protections; (6) apply product deployment, management, and maintenance best practices; (7) publish trial results inclusive of clinically meaningful outcomes in peer-reviewed journals; (8) have results reviewed and cleared or approved by regulatory bodies as required to support product claims of risk, efficacy, and intended use; (9) make claims appropriate to clinical validation and regulatory status; and (10) collect, analyze, and apply real-world evidence and product performance data [10].

**Figure 1:**
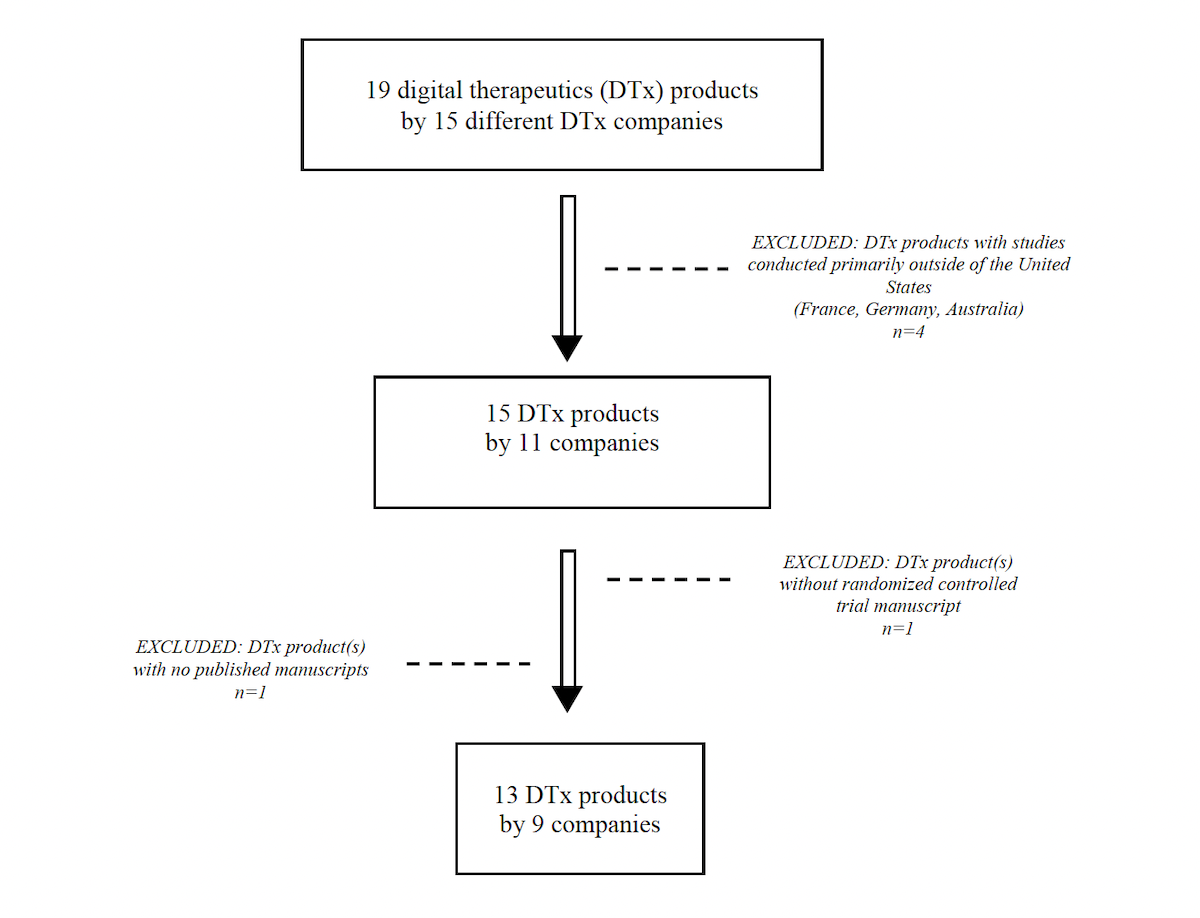
Digital Therapeutics Alliance database DTx product search strategy.

At the time of this analysis, there were 19 DTx products by 15 DTx companies listed in the DTA Product Library. DTx products created in the United States were prioritized for our review due to the availability of demographic data (eg, race and ethnicity) and diverse composition of the US population. DTx products with studies conducted primarily outside of the United States (n=4), abstracts without peer-reviewed manuscripts (n=1), or DTx products without an RCT manuscript (n=1) were excluded in this analysis (see Figure 1). Within the DTA Product Library, we reviewed the Clinical Trials section of each of DTx products. We obtained titles of studies published for each DTx product and used the PubMed database to retrieve supporting manuscripts. For each DTx product, we reviewed manuscripts with RCTs as the main study design; review of additional manuscripts was conducted and considered independently if there were multiple manuscripts published supporting a DTx product. However, those findings are not presented here as the focus of this paper is on RCTs. The titles and abstracts of each study were reviewed before data extraction.

### Data Analysis

The following demographic information was retrieved from RCTs meeting inclusion criteria outlined above: age, sex, race, ethnicity, education, marital status, employment, and insurance type. These demographics were selected as they are routinely collected and analyzed in studies, have been deemed necessary to be included in National Institutes of Health (NIH)–sponsored studies, and are typically linked with potential differential outcomes in studies [11]. Study demographics were extracted regardless of whether the information was in a demographics table or in the body of the manuscript. We used 4 categories for race: “Asian,” “Black,” “White,” and “Other” as the reporting in studies was most consistent in this manner. For ethnicity, that is, Hispanic or non-Hispanic participants, there was 1 manuscript that reported the Hispanic group as being combined with Black/African American. We make a note of this in Tables 1-4. Characteristics extracted from the included studies are presented as counts (percentages) or means (SD). Several studies provided only sample size and percentage data. We used the sample size and percentage data to estimate the number of participants per demographic domain, for example, sex, race, and ethnicity counts. Sex, race, and ethnicity were compared with the distribution of the US population to quantify sex and minority representation in the study [12]. Studies with lower percentages than the population distribution for racial and ethnic groups (eg, Asian <5·6%, Black <13·4%, Hispanic <18·5%) or sex (eg, female <50·8%) were considered to be underrepresented in the respective study, an approach that has been used previously [13].

**Table 1:** DTx^a^ clinical trial demographic data from the Digital Therapeutics Alliance Product Library: reSet-0, Reset, FreeSpira, and Deprexis.

Digital Therapeutic Product				reSet-0		Reset		FreeSpira		Deprexis
Company				Pear Therapeutics		Pear Therapeutics		FreeSpira Inc		Orexo
FDA^b^ regulation				FDA cleared		FDA-cleared class II medical device		FDA cleared		—^c^
Study type				Randomized controlled trial		Randomized controlled trial		Randomized controlled trial		Randomized controlled trial
Name of study				Adding an Internet-delivered treatment to an efficacious treatment package for opioid dependence		Internet-delivered treatment for substance abuse: a multisite randomized controlled trial		Feedback of end-tidal pCO2 as a therapeutic approach for panic disorder		Effectiveness of an internet intervention (Deprexis) for depression in a United States adult sample: a parallel-group pragmatic randomized controlled trial
Digital therapeutic clinical area of focus				Opioid use disorder		Substance use disorder		Panic disorder/PTSD^d^		Depression
Prescription required?				Yes		Yes		Yes		No
**Demographics**										
	**Sample size, n**		170		507		37		376	
	Age (years)		Mean 34.3 (SD 9.9)		Mean 34.9 (SD 10.9)		Range 18-60		Mean 31.9 (SD 11.2)	
	**Sex, n (%)**									
		Male	92 (54.1)		315 (62.1)		13 (35.0)		93 (24.7)	
		Female	78 (45.9)		192 (37.9)		24 (65.0)		280 (74.4)	
	**Race, n (%)**									
		Asian	0 (0)		13 (2.6)		3 (8.1)		27 (7.2)	
		Black	0 (0)		116 (22.9)		1 (2.7)		15 (3.9)	
		White	162 (95.3)		284 (56.0)		32 (86.5)		291 (77.3)	
		Others	8 (4.7)		92 (18.1)		0 (0)		3 (0.8)	
	**Ethnicity, n (%)**									
		Hispanic	N/A		55 (10.8)		1 (2.7)		42 (11.1)	
		Non-Hispanic	—		452 (89.2)		36 (97.3)		325 (86.4)	
	Education: some college or more		Median 12 (IQR 12-14)^e^		n=79 (15.6%)		—		n=324 (86.2%)	
	Employment: employed at least half time n (%)		62 (36.6)		317 (62.5)		—		—	
	Married, remarried, or partnered, n (%)		Never married: 44^f^		72 (14.2)		22 (59.5)		71 (19.1)	
	Percentage of participants with private insurance (%)		—		100		—		—	

^a^DTx: digital therapeutics.

^b^FDA: Food and Drug Administration.

^c^Data not available in study.

^d^PTSD: posttraumatic stress disorder.

^e^Study reported education as either mean years with range or median with IQR.

^f^Study only reported number and percentage of “never married” individuals for marriage status.

**Table 2:** DTx^a^ clinical trial demographic data from the Digital Therapeutics Alliance Product Library—Propeller, Bluestar (randomized controlled trial), Bluestar (secondary analysis of the randomized controlled trial), and d-Nav.

Digital Therapeutic Product					Propeller			Bluestar			Bluestar			d-Nav
Company					Propeller Inc			WellDoc			WellDoc			Hygeia
FDA^b^ regulation					FDA-cleared Class II Medical Device			FDA-cleared Class II Medical Device			FDA-cleared Class II Medical Device			FDA-cleared Class II Medical Device
Study type					Randomized controlled trial			Randomized controlled trial			Secondary analysis of a cluster randomized controlled trial^c^			Randomized controlled trial
Name of study					A randomised controlled trial of the effect of a connected inhaler system on medication adherence in uncontrolled asthmatic patients			WellDoc mobile diabetes management randomized controlled trial: change in clinical and behavioral outcomes and patient and physician satisfaction			Mobile diabetes intervention for glycemic control: impact on physician prescribing			Automated insulin dosing guidance to optimise insulin management in patients with type 2 diabetes: a multicentre, randomised controlled trial
Digital therapeutic clinical area of focus					COPD^d^/asthma			Type 1/type 2 diabetes			Type 1/type 2 diabetes			Type 2 diabetes
Prescription required?					No			No			No			Yes
**Demographics**														
	Sample size, n			437			26			117			181	
	Age (years)			Mean 47 (SD 15.0)			Range 20-64			Mean 52.7 (SD 8.2)			Mean 60.3 (SD 7.9)	
	**Sex, n (%)**													
		Male	153 (35.0)			9 (34.6)			59 (50.4)			93 (51.4)		
		Female	284 (65.0)			17 (65.4)			58 (49.6)			88 (48.6)		
	**Race, n (%)**													
		Asian	22 (5.0)			0 (0)			0 (0)			7 (3.9)		
		Black	34 (8.0)			10 (38.5)			43 (36.8)			38 (20.9)		
		White	373 (85.0)			16 (61.5)			65 (55.6)			125 (69.1)		
		Others	0 (0)			0 (0)			9 (7.7)			11 (6.1)		
	**Ethnicity, n (%)**													
		Hispanic	—^e^			—			—			8 (4.4)		
		Non-Hispanic	—			—			—			145 (80.1)		
	Education: some college or more, n (%)			—			—			85 (72.6)			—	
	Employment: employed at least half time n (%)			—			—			—			—	
	Married, remarried, or partnered, n (%)			—			—			—			—	
	Percentage of participants with private insurance (%)			—			100			100			—	

^a^DTx: digital therapeutics.

^b^FDA: Food and Drug Administration.

^c^Study reported Hispanic Ethnicity and Black/African American as one group under Race Demographic Category.

^d^COPD: chronic obstructive pulmonary disease.

^e^Data not available in study.

**Table 3:** DTx^a^ clinical trial demographic data from the Digital Therapeutics Alliance Product Library—Leva, Propeller, Daylight, and EndeavorRx.

Digital Therapeutic Product				Leva		Propeller		Daylight		EndeavorRx
Company				Renovia		Propeller Inc		Big Health		Akili Interactive
FDA^b^ regulation				FDA-cleared Class II Medical Device		FDA-cleared Class II Medical Device		—^c^		FDA-authorized Class II Medical Device
Study type				Randomized controlled trial		Randomized controlled trial		Randomized controlled trial		Randomized controlled trial
Name of study				Multicenter randomized controlled trial of pelvic floor muscle training with a motion-based digital therapeutic device versus pelvic floor muscle training alone for treatment of stress-predominant urinary incontinence		The impact of patient self-monitoring via electronic medication monitor and mobile app plus remote clinician feedback on adherence to inhaled corticosteroids: a randomized controlled trial		Efficacy of digital cognitive behavioral therapy for moderate‐to‐severe symptoms of generalized anxiety disorder: a randomized controlled trial		A novel digital intervention for actively reducing severity of paediatric ADHD (STARS-ADHD): a randomised controlled trial
Digital therapeutic clinical area of focus				Female urinary incontinence		COPD^d^/asthma		Generalized anxiety disorder		ADHD^e^
Prescription required?				Yes		No		No		Yes
**Demographics**										
	Sample size, n		77		100		256		348	
	Age (years), mean (SD)		52.1 (14.0)		48.5 (12.3)		30.9 (10.7)		9.7 (1.3)	
	**Sex, n (%)**									
		Male	0 (0)		20 (20.0)		77 (30.1)		248 (71.3)	
		Female	77 (100.0)		80 (80.0)		179 (69.9)		100 (28.7)	
	**Race, n (%)**									
		Asian	0 (0)		6 (6.0)		7 (2.7)		—	
		Black	10 (13.0)		26 (26.0)		10 (3.9)		—	
		White	52 (67.5)		68 (68.0)		215 (84.0)		—	
		Others	8 (10.4)		0 (0)		24 (9.4)		—	
	**Ethnicity, n (%)**									
		Hispanic	5 (6.5)		5 (5.0)		—		—	
		Non-Hispanic	72 (93.5)		95 (95.0)		—		—	
	Education: some college or more, n (%)		—		87 (87)		212 (82.8)		—	
	Employment: employed at least half time, n (%)		—		—		—		—	
	Married, remarried, or partnered, n (%)		—		—		112 (43.6)		—	
	Percentage of participants with private insurance		—		—		—		—	

^a^DTx: digital therapeutics.

^b^FDA: Food and Drug Administration.

^c^Data not available in study.

^d^COPD: chronic obstructive pulmonary disease.

^e^ADHD: attention-deficit hyperactivity disorder.

**Table 4:** DTx^a^ clinical trial demographic data from the Digital Therapeutics Alliance Product Library—Sleepio, Somryst, and Nerivio.

Digital Therapeutic Product				Sleepio		Somryst		Nerivio
Company				Big Health		Pear Therapeutics		Theranica
FDA^b^ regulation				—^c^		FDA-cleared class II medical device		De novo FDA-authorized
Study type				Randomized controlled trial		Randomized controlled trial		Prospective, randomized, double-blind, placebo-controlled, multicenter trial^d^
Name of study				Effect of digital cognitive behavioral therapy for insomnia on health, psychological well-being, and sleep-related quality of life: a randomized clinical trial		Effect of a web-based cognitive behavior therapy for insomnia intervention with 1-year follow-up: a randomized clinical trial		Remote electrical neuromodulation (REN) relieves acute migraine: a randomized, double-blind, placebo-controlled, multicenter trial
Digital therapeutic clinical area of focus				Insomnia		Chronic insomnia		Migraine
Prescription required?				No		Yes		Yes
**Demographics**								
	Sample size, n			1711		303		252
	Age (years), mean (SD)			48.1 (13.8)		43.3 (11.6)		43 (12.0)
	**Sex, n (%)**							
		Male	382 (22.3)		85 (28.1)		48 (19.0)	
		Female	1329 (77.7)		218 (71.9)		204 (81.0)	
	**Race, n (%)**							
		Asian	45 (2.6)		12 (4.0)		0 (0)	
		Black	19 (1.1)		21 (6.9)		0 (0)	
		White	1558 (91.1)		254 (83.8)		221 (87.7)	
		Others	88 (5.1)		16 (5.3)		31 (12.3)	
	**Ethnicity, n (%)**							
		Hispanic	—		24 (7.9)		—	
		Non-Hispanic	—		279 (92.1)		—	
	Education: some college or more			Mean 16.6 years of continuous education^e^		n=235 (77.6%)		—
	Employment: employed at least half time, n (%)			1152 (67.3)		175 (57.8)		—
	Married, remarried, or partnered, n (%)			—		181 (59.7)		—
	Percentage of participants with private insurance			—		—		—

^a^DTx: digital therapeutics.

^b^FDA: Food and Drug Administration.

^c^Data not available in study.

^d^Study reported Hispanic Ethnicity and Black or African American as one group under Race Demographic Category.

^e^Study reported education as either mean years with range or median with IQR.

### Ethical Considerations

We did not apply for ethics approval as this study is a secondary analysis and report of publicly available manuscript data.

## Results

After applying exclusion criteria, there were 10 DTx companies with 13 DTx products remaining for analysis. We examined 15 studies across various health domains (ie, diabetes, sleep or insomnia, migraine, depression, substance use disorders, generalized anxiety disorder, panic disorder, posttraumatic stress disorder, attention-deficit/hyperactivity disorder, chronic obstructive pulmonary disorder, and female urinary incontinence).

Study sample sizes ranged from 26 to 1711 with a median study size of 252 (IQR 109-362). A total of 13 of the 15 (87%) studies reported mean age. The median of the reported mean ages is 43.3 (IQR 34.3-48.5) years. The remaining 2 studies did not report mean age, only the age range of participants. All studies reported sex status with 10 of 15 (67%) studies reporting that females made up ≥65% of the study cohort. Of note, 1 of the 10 studies reported 100% women as the DTx product is aimed specifically at female urinary incontinence. Two studies had roughly equal representation of males and females. Of 14 studies that reported race data, Asian individuals were underrepresented in 11 (79%) studies and Black or African American individuals in 9 (64%) studies. A total of 7 of the 15 (47%) studies reported ethnicity; Hispanics were underrepresented in 7 (100%) of these studies. Similarly, study participants’ education levels were not consistently reported. Some studies reported mean years of education, whereas others provided counts, percentages, or median years of education. A total of 8 (53%) studies reported education levels, 5 of which had populations in which 70% or more had at least some college education. In addition, 1 study reported a mean of 16.6 years of education. Another study reported a median of 12 (IQR 12-14) years. Only 4 of the 15 (27%) studies reported employment information, with 3 of the 4 (75%) consisting of study populations in which a majority, ≥57% of participants, were employed part-time or full-time. Only 3 (20%) studies reported insurance information, with each of these studies reporting a study cohort in which 100% of members were privately insured.

## Discussion

### Principal Findings

To our knowledge, this is the first study to review DTx literature for demographic representation in clinical trials. As DTx products are increasingly being marketed as a potential equalizer for medically underserved communities, it is important to assess the clinical trials population basis for the development, testing, and validation of these products to ensure uptake, proper use, and efficacy of DTx solutions in all populations, especially medically vulnerable populations.

Our analysis of the DTx RCT literature demonstrates mixed findings among different dimensions of study demographics. Broadly, our findings indicate that the average participant in DTx trials is a well-educated, middle-aged, female with private health insurance; similar findings have been documented elsewhere regarding telehealth usage [14]. With the majority of individuals being, on average, 43 years old, this does not adequately reflect the aging population that continues to grow as the management of chronic disease improves. The relative abundance of women represented in DTx clinical trials proves hopeful and counters the traditional sex imbalance seen in years past. However, it is important to continue to ensure balanced populations, more closely reflective of the US sex demographics where necessary (ie, DTx can be used across multiple sexes). A recent review of clinical trials by Buffenstein et al [15] looked at 44 different diseases from 2008 to 2019. It found that only 53% of trials reported information on race and 36% reported information on ethnicity. Another review by Duma et al [16] focused on cancer studies from 2003 to 2016 and found that only 31% reported information on ethnicity. In contrast, our study on DTx clinical trials shows better reporting rates, with 93% reporting race and 47% reporting ethnicity.

However, similar to Buffenstein et al’s [15] review, our study found that certain groups were still underrepresented in DTx trials. Asian individuals, Black individuals, Hispanic individuals, and older adults had lower participation rates than their representation in the general population. This aligns with previous reviews that showed similar patterns of underrepresentation. On the other hand, there continues to be a higher representation of females in DTx trials.

Further, in a review of rheumatoid arthritis trials from 2008 to 2018, Strait et al [17] found lower participation rates among racial and ethnic minorities than their representation in the US Census population. They concluded that there was no improvement in representing these groups. Similarly, an examination of Pfizer’s clinical trials from 2011 to 2020 found that while Black and female enrollment matched census levels, Hispanic populations remained underrepresented [18].

These reviews across different disease categories, including outside the DTx field, indicate that efforts are being made to increase representation among diverse groups. However, more work needs to be done. Our findings in DTx trials support this trend, highlighting the need for better recruitment of racial and ethnic minorities, including Asian and Hispanic individuals, as well as individuals from different age groups and SES.

Our findings also indicate disproportionate representations of racial, ethnic, and socioeconomic diversity—that is, education, employment, and insurance—in DTx clinical trial cohorts. These findings are congruent with existing literature on the paucity of several dimensions of diversity in clinical trials [19]. This finding is concerning as the usability of technology decreases with education levels and age. Budding literature suggests differential use of digital health tools based on age, sex, SES, marital status, and geography, further highlighting the need to better understand the complex sociotechnical interactions between different communities and these promising tools at the onset of development and testing [20-22]. Thus, equity-centered approaches to digital health innovations such as the digital health equity framework proposed by Crawford and Serhal [23] and others [14,19,24] are recommended.

As DTx products continue to proliferate, we recommend the DTA, the leading not-for-profit advocacy group championing DTx, to consider adding an 11th foundational principle, making explicit diversity in clinical trials a best practice of DTx, whenever and wherever applicable. DTA should borrow language from the NIH Guidelines on the Inclusion of Women and Minorities as Subjects in Clinical Research [11] to ensure adequate representation where necessary and encourage researchers to justify why populations are not included in cases where it may be inappropriate to do so. By adding diversity in trials as a core principle, the DTA can help to ensure that these technologies are designed and implemented for the equitable benefit of diverse communities. Without an intentional, inclusive design approach to DTx, it may be difficult to actualize the potential of these emerging therapies to reach vulnerable populations as has been suggested by others [25].

Finally, to assess and ensure adequate representation, it is important that DTx clinical trial studies be consistent in the reporting of key demographic information. Sociodemographic data, especially in clinical trials, are critical for conducting subgroup analyses to further elucidate complex therapeutic and sociotechnical interactions [7,19,24]. Like all other RCTs, diversity in DTx RCT populations is a foundation for promoting equity in these emergent therapies.

### Limitations

The limitations of this study include the use of a small, focused database. Consequently, the generalizability of this study to the broader field of emerging DTx clinical trials is limited. This is partly due to the difficulty of finding accessible, comprehensive databases of quality DTx products, especially at this early stage of development. While the US National Library of Medicine’s ClinicalTrials.gov remains a useful resource for searching completed DTx trials, the DTx products featured in the DTA Product Library are a unique set of products [26]. These products have been developed by companies that have signed the DTA pledge, which acknowledges their commitment to developing quality, reliable products with inclusive design and trials that produce clinically meaningful results. This makes it possible to conduct more comparable analyses across the studies in the DTA database. Although small, there are benefits to the use of the DTA Product Library as the companies represented have agreed to shared DTx best practices regarding product development, user engagement, and rigorous testing. Additionally, we also focused primarily on RCTs that may have limited the data available for analysis as DTx is a nascent field. However, our primary focus on RCTs and the resulting findings further emphasizes the need for more diverse clinical trial research cohorts as RCTs are the gold standard and readily inform clinical practice.

### Conclusions

Our findings build on previously stated concerns regarding representativeness of study populations during development and evaluation of digital health tools. This study provides data that illuminate opportunity within an advantageous window. With DTx in its infancy, DTx companies have the chance to be even more inclusive in the development, testing, and subsequent access to these products as a preventative measure to promote and assure health equity. To design more inclusive DTx clinical trials, companies could implement several strategies. For example, targeted recruitment could involve partnering with community-based organizations and patient advocacy groups to identify potential trial participants from underrepresented populations or using social media and community events to reach out to these groups. To ensure a socioeconomically diverse participant pool, companies could consider factors such as access to care and technology, including providing participants with necessary devices or internet access and offering flexible scheduling for study visits. Furthermore, actively reporting and monitoring diversity metrics throughout the trial, including representation across race, ethnicity, sex, and SES, would allow companies to adjust recruitment strategies as needed and ensure that the trial represents the intended patient population. This proactive approach would help promote the widespread benefits of emerging DTx therapies while ensuring equitable access to these innovative solutions.

In all, inclusive research cohorts ensure that technological advancements in care delivery do not introduce further disparities, creating new goal posts for underserved populations. By taking a more proactive and prospective approach, the DTx community can help prevent the further deepening of the digital divide and aide in developing solutions that invariably promote equitable outcomes.

## Abbreviations

DTA: Digital Therapeutics Alliance
DTx: digital therapeutics
NIH: National Institutes of Health
RCT: randomized controlled trial
SES: socioeconomic status

## Data Availability

All data used in this study are presented in Tables 1-4 along with the names of the studies for follow-up.
